# Design of stable circular permutants of the GroEL chaperone apical domain

**DOI:** 10.1186/s12964-023-01426-4

**Published:** 2024-02-01

**Authors:** Tatiana N. Melnik, Maria A. Majorina, Daria E. Vorobeva, Galina S. Nagibina, Victoria R. Veselova, Ksenia A. Glukhova, Marina A. Pak, Dmitry N. Ivankov, Vladimir N. Uversky, Bogdan S. Melnik

**Affiliations:** 1 Institute of Protein Research, Russian Academy of Sciences, Institutskaja Str. 4, Pushchino, Moscow Region 142290 Russia; 2 Institute of Theoretical and Experimental Biophysics, Russian Academy of Sciences, Institutskaja Str. 3, Puschino, Moscow Region 142290 Russia; 3 Center for Molecular and Cellular Biology, Skolkovo Institute of Science and Technology, Bolshoy Boulevard 30, Bld. 1, Moscow, 121205 Russia; 4 Department of Molecular Medicine and USF Health Byrd Alzheimer’s Center and Research Institute, Morsani College of Medicine, University of South Florida, Tampa, FL USA; 5 Research Center for Molecular Mechanisms of Aging and Age-Related Diseases, Moscow Institute of Physics and Technology, Dolgoprudny, Russia; 6 Pushchino Branch, Shemyakin–Ovchinnikov Institute of Bioorganic Chemistry, Russian Academy of Sciences, Prospekt Nauki 6, Pushchino, Moscow Region 142290 Russia

## Abstract

Enhancing protein stability holds paramount significance in biotechnology, therapeutics, and the food industry. Circular permutations offer a distinctive avenue for manipulating protein stability while keeping intra-protein interactions intact. Amidst the creation of circular permutants, determining the optimal placement of the new N- and C-termini stands as a pivotal, albeit largely unexplored, endeavor. In this study, we employed PONDR-FIT’s predictions of disorder propensity to guide the design of circular permutants for the GroEL apical domain (residues 191–345). Our underlying hypothesis posited that a higher predicted disorder value would correspond to reduced stability in the circular permutants, owing to the increased likelihood of fluctuations in the novel N- and C-termini. To substantiate this hypothesis, we engineered six circular permutants, positioning glycines within the loops as locations for the new N- and C-termini. We demonstrated the validity of our hypothesis along the set of the designed circular permutants, as supported by measurements of melting temperatures by circular dichroism and differential scanning microcalorimetry. Consequently, we propose a novel computational methodology that rationalizes the design of circular permutants with projected stability.

Video Abstract

**Supplementary Information:**

The online version contains supplementary material available at 10.1186/s12964-023-01426-4.

## Introduction

Designing proteins with desired properties [1, 2] is of paramount importance in biotechnology, medicine, and the food industry [3–5]. One sought-after modification is the enhancement of protein stability without compromising protein function [5, 6]. Conventional approaches involve the introduction of single- and multi-point mutations to improve protein stability [6]. In cases where protein N- and C-termini are located close to each other, circular permutations can also serve as a method to modify protein stability and the mobility of specific protein regions [7–13].

Two factors are critical for the successful design of circular permutants (CPs): (i) the structure and length of the linker connecting the original N- and C-terminal amino acid residues of the protein, and (ii) the cleavage site for generating new N- and C-termini. While numerous studies have concentrated on linker design [14–16], the selection of the circular permutation site has garnered limited attention in existing literature. Typically, experimentalists choose a cleavage site based on intuition and personal experience [17]. Consequently, the site's placement ends up being either nearly arbitrary or involves testing multiple potential options. This methodology necessitates the isolation and purification of each proposed circular permutant, followed by an assessment of its stability and activity [8], which is time-consuming and labor-intensive. Hence, the development of computational strategies for designing novel N- and C-termini in circular permutants is of great significance.

In an earlier work, we employed predictive tools [18, 19] for identifying intrinsically disordered regions to pinpoint optimal sites for introducing disulfide bonds aimed at enhancing protein stability [20, 21]. Analysis of the compact structures of globular proteins using these prediction methods [18, 19] unveiled that almost all proteins contain small regions that are intrinsically disordered, even in the presence of their well-defined structures, as observed in crystallographic or NMR studies. Drawing from this insight, we effectively engineered cysteine bridges to increase the stability of GFP [20] and GaO protein [21]. Furthermore, an exploration of L1 ribosomal proteins across diverse organisms highlighted the potential of PONDR-family programs in identifying weakened regions within proteins exhibiting similar three-dimensional (3D) structures [22–24].

In this research, we employed predictions from the PONDR® VLXT program to create circular permutations of the apical domain of the GroEL (AD-GroEL) chaperone [25], each exhibiting distinct stability. The GroEL apical domain plays an important role in binding non-native target proteins [26–31], a critical aspect of the GroEL chaperone's functionality. When examined in isolation, the 15 kDa GroEL apical domain retains some of its functional characteristics, enabling it to bind non-native proteins, and is often denoted as a "minichaperone" [28, 32–35]. Given the proximity of the N- and C-termini within the GroEL apical domain (residues 191–335) [25], it stands as an apt model for investigating circular permutations.

We postulate that a decreased degree of disorder at the chosen cleavage site indicates increased stability for the corresponding CP. To investigate this conjecture, we designed six CPs of the GroEL apical domain utilizing glycine residues as cleavage sites. The designed CPs, in conjunction with the wild-type protein, exhibited a broad spectrum of stability, as assessed through circular dichroism (CD) and differential scanning microcalorimetry (DSC). The melting temperatures (T_m_) of these proteins displayed a reasonable correlation with the predictions generated by the PONDR® VLXT program, yielding Pearson correlation coefficients ranging from 0.66 to 0.92, contingent on the experimental method employed to define T_m_. These findings underscore the feasibility of the proposed strategy for engineering CPs with desired stability, establishing a robust computational framework for this objective.

## Materials and methods

### Protein expression and purification

The expression vectors were obtained by cloning DNA fragments encoding the *Escherichia coli* GroEL apical domain (amino acids 192–333 of GenBank protein sequence no. NP_418567.1) or its circular permutants into the plasmid vector pET11cjoe. DNA fragments were produced by polymerase chain reaction (PCR) from the plasmid template pET11cjoe GroES-GroEL [36]. Circular permutations were made by three separate PCRs. The first PCR amplified the 3′ end of the GroEL apical domain cDNA (encoding amino acids 210–333, 244–333, 256–333, 269–333, 282–333, or 297–333). The forward primer contained an NdeI restriction site, and the reverse primer included a three-glycine linker coding sequence. The second PCR amplified the 5′ end of the GroEL apical domain cDNA (encoding amino acids 192–209, 192–243, 192–255, 192–268, 192–281, or 192–296) with the forward primer coding for three-glycine linker and the reverse primer containing a stop codon followed by a SalI restriction site. The PCR products were purified by agarose gel electrophoresis; corresponding pairs were mixed and used as a template for the third PCR with primers containing restriction sites. The final PCR products were purified and cloned into the plasmid vector using NdeI and SalI sites.

GroEL apical domain and its circular permutant forms were expressed in *E.coli* cells and isolated as described elsewhere [37] with some modifications. The purity of the isolated protein was checked by the SDS-PAGE.

### Polyacrylamide gel electrophoresis (PAGE) under native conditions

The native PAGE experiment was performed with Mini PROTEIN®System (BIO-RAD, USA), under the following conditions: 15% (w/v) separating gel with a 4% (w/v) stacking gel (non-denaturing polyacrylamide gel) in Tris–glycine (pH 7.2), and 6-µL samples were loaded. The proteins were stained with Coomassie Blue G250.

### Circular dichroism (CD) spectroscopy

The Far-UV CD spectra and ellipticity dependence on temperature were measured using a JASCO–1500 spectropolarimeter (Japan Spectroscopic Co, Japan) equipped with a temperature-controlled holder in 0.1 mm thick cells at the protein concentration of 0.1–0.4 mg/ml. The molar ellipticity [θ] was calculated from the equation, [θ] = (θ_obs_*M_res_)/(c*L) where *c* is the protein concentration (g/l), *L* is the optical path length of the cell (mm), θ_obs_ is the ellipticity measured (in degrees) at wavelength *λ* (nm), and *M*_res_ is the mean residue molecular mass of the protein. The dependence of protein ellipticity on temperature was carried out in a 10 mM sodium phosphate buffer, pH = 7.2 in the temperature range from 20° C to 90° C in 1°C increments. The Far-UV CD spectra were measured at 20° C.

The protein concentrations were determined by the method of Waddel based on the difference between spectrophotometric absorption at 215 nm and 225 nm [38].

### Differential scanning calorimetry (DSC)

DSC measurements were made on a precision scanning microcalorimeter SCAL-1 (Scal Co., Ltd., Russia) with 0.33 ml glass cells [39] at a scanning rate 1 °C per min and under the pressure of 2.5 atm. The experiments were performed between 0 and 100 °C in 20 mM sodium phosphate buffer at pH 7.2. The protein concentrations in the experiment varied from 1.9 mg/ml to 5.5 mg/ml. The experimental DSC profiles were corrected for the calorimetric baseline, and the molar partial heat capacity functions were calculated in a standard manner. The excess heat capacity was evaluated by subtracting the linearly extrapolated initial and final heat capacity functions with correction for the difference of these functions by using a sigmoid baseline [40]. A typical value for the partial specific volume for globular proteins (0.73cm3/g) was accepted arbitrarily, since it does not influence the calculated excess heat capacity.

## Results and discussion

### Analysis of circular permutant amino acid sequences

In this study, we examine the hypothesis that the location for the new N- and C-termini within a circular permutant, intended to impact protein stability, can be chosen based on predictions of intrinsic disorder scores. According to this hypothesis, regions with lower intrinsic disorder scores will experience minimal destabilization upon chain cleavage, primarily due to the robust stabilizing effect of intra-protein interactions within such areas. Consequently, the impact of chain cleavage on protein stability in these regions is expected to be minimal, as the new N- and C-termini are less likely to undergo significant fluctuations. Conversely, amino acid residues characterized by high disorder values suggest weaker stabilization within the corresponding protein region. Thus, cleaving the chain at such a location is predicted to lead to substantial fluctuation of the new N- and C-termini, resulting in the loss of stabilizing intra-protein contacts and configurational entropy increase. As a result, we expect an inverse correlation between the predicted degree of disorder at the cleavage site prior to circular permutation and the stability of the resulting circular permutant.

**Fig. 1:**
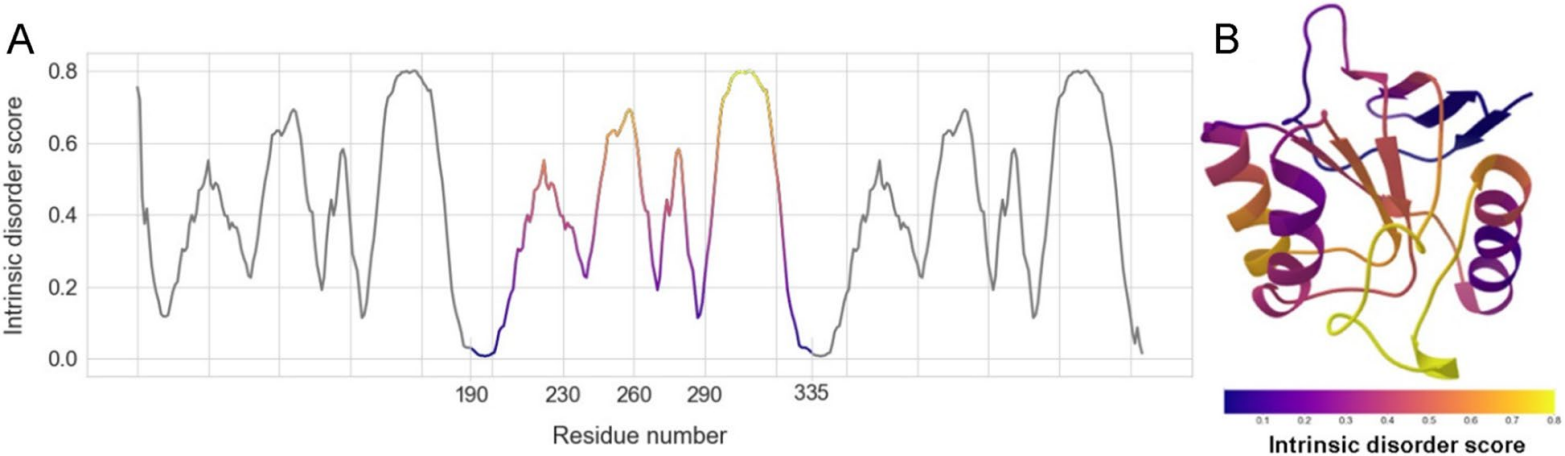
**A** Intrinsic disorder profile of AD-GroEL (amino acid residues from 191 to 335 of the full-length GroEL) predicted by PONDR® VLXT. The sequence is replicated three times; the central fragment can be considered as a circular amino acid sequence of AD-GroEL. **B** The 3D structure of AD-GroEL colored according to the PONDR® VLXT predictions from (**A**)

The design of a circular permutant involves initially connecting the original N- and C-termini of a protein. Subsequently, a new cleavage site for this circular protein must be chosen, guided by disorder predictions. However, it is important to note that the PONDR® VLXT program [41] (and, as a matter of fact any other algorithms for prediction of the per-residue disorder propensity) was not specifically designed for the analysis of circular protein sequences. It processes linear protein chains and consequently assigns additional disorder to the N- and C-terminal regions. To circumvent the influence of protein termini and accurately determine the disorder level of the circular amino acid sequence, we replicated the amino acid sequence of the GroEL apical domain threefold (Fig. 1) and conducted the calculation using the PONDR® VLXT program. The outcomes obtained for the central fragment of this triplicated sequence can be leveraged to gauge the disorder extent of amino acid residues within a circular permutant.

### Selecting sites for circular permutations

Aside from the placement of the new N- and C-termini, several factors can potentially influence the stability of circular permutants, such as the specific amino acid residues at the new termini and their corresponding secondary structure within the wild-type protein. To alleviate the potential impact of these factors, we focused on loop regions containing glycine residues as potential cleavage sites. This decision is considered judicious because it minimizes the likelihood of disrupting the existing secondary structure elements.

**Fig. 2:**
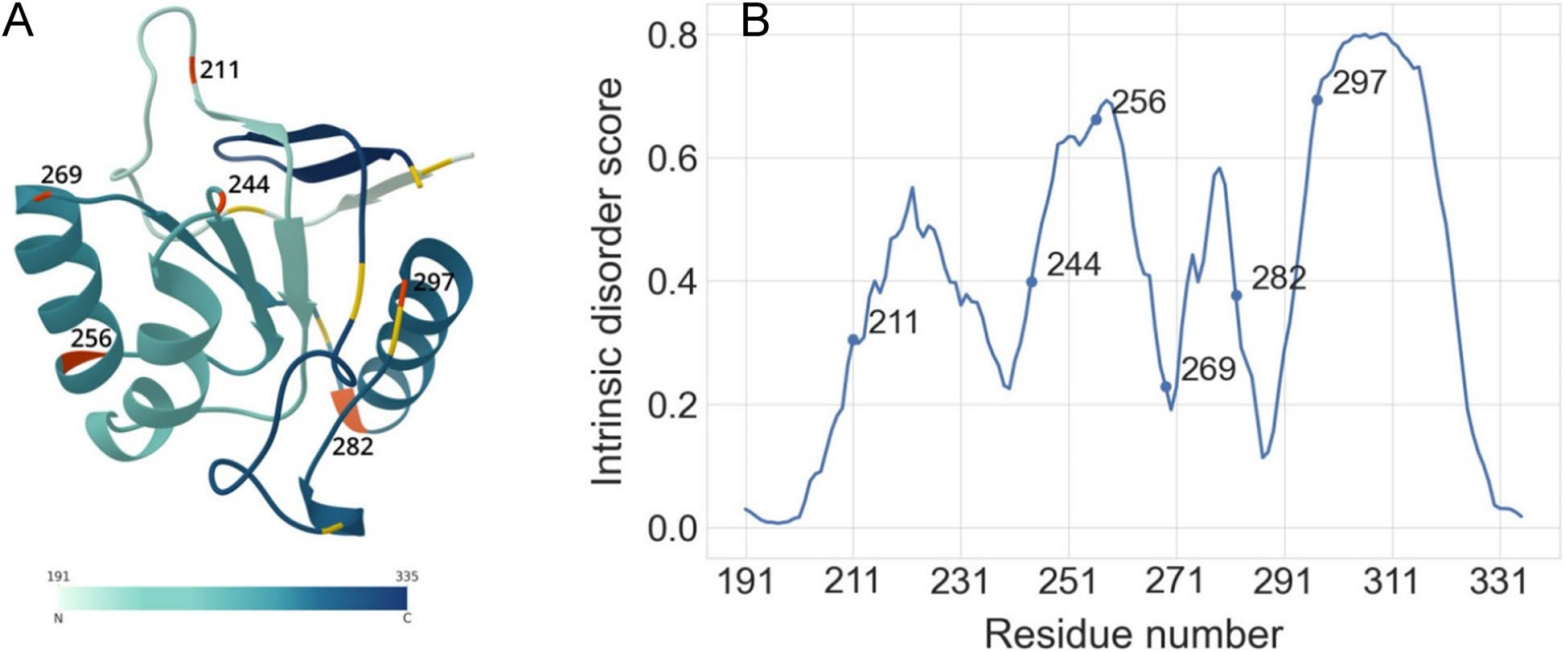
**A** 3D structure of AD-GroEL (PDB: 1DER, residues from 191 to 335). The numbering of amino acids corresponds to the numbering in the structure of a full-length protein. The protein is colored from N- to C- terminus according to the color scale at the bottom of the Figure. Thirteen glycines are denoted as orange or dark grey. The six glycines selected as cleavage points are colored in orange and are labeled by their residue number. The other glycines are shown in dark grey. **B** Probability of intrinsic disorder of the AD-GroEL circular sequence calculated by the PONDR® VLXT program. The circles denote the glycines at the site of the designed breaks in the amino acid sequence

We identified all thirteen glycine residues within the apical domain of GroEL and specifically chose those situated within loops, while excluding glycines located in regions critical for maintaining the regular secondary structure of the protein. Additionally, glycines positioned at the N- and C-terminal regions were not considered. In cases where two glycines were situated in close proximity, only one of them was chosen arbitrarily for further analysis. Consequently, we identified six glycine residues at positions 211, 244, 256, 269, 282, and 297 within the GroEL apical domain as suitable candidates for circular permutation and the subsequent design of new N- and C-termini (Fig. 2).

Making a cut within a loop of the original protein to generate new ends in the permutein makes perfect sense because of the two reasons. First, this ensures that the important elements of regular secondary structure are minimally affected. Second, it is known that the intrinsic disorder is unevenly distributed within the hybrid proteins containing both ordered and disordered regions, being more commonly found at the N- and C-termini of a typical globular protein [42]. Therefore, the new N- and C-termini produced by a cut within the disordered or flexible loop will be disordered or flexible in native conditions, as practically always happens in globular proteins [42].

In accordance with our hypothesis, there should be an inverse correlation between the stability of circular permutants and the level of disorder at the cleavage site. Therefore, based on the results obtained from calculations using the PONDR® VLXT program (Fig. 2B), among the six glycine residues we selected, the chain cleavage in proximity to Gly269 or Gly211 is expected to yield the highest stability (Fig. 2). Conversely, the circular permutants with a chain cleavage near Gly297 or Gly256 is projected to exhibit the greatest degree of destabilization.

We recognize that one could maintain the opposite hypothesis, where cutting a higher disorder region would be less disruptive (less destabilizing) than cutting an ordered region. However, in reality, it is difficult to give a solid answer to a question if a region with high disorder level is good or bad for mutations. In fact, after conducting a lot of research on the effect of different mutations on the folding of globular proteins (see for example [43]), we came to the following conclusions. In an ordered protein, there is a very limited number of contacts inside a "weak region" (i.e., a region with higher disorder score) that stabilize it. Breaking of even a very few of these contacts might cause big changes in protein stability. If the same is done in a well-stabilized region (i.e., a region with low disorder score), the corresponding mutation will disrupt a small number of exiting interactions, and in general, will not have a very strong effect on the protein stability. We are using similar arguments here, while considering design of circular permutants. We also recognize that the reality is much more complex, as there are important dependencies on the secondary structure of the protein, its domain structure, and many other factors. Although it is unlikely that a strong correlation between the disorder status of a region subjected to mutation and the overall protein stability would be unequivocally established, it seems that there is a working "recipe" for the researchers. The “formula” is supported by the outputs of our three studies in this direction. First, we established that it was possible to evaluate the effects of single mutations on protein stability using PONDR [24, 44]. Then, we showed that this technique can be used for the design of stabilizing cysteine bridges [20, 21, 23, 24]. And now, we are showing the applicability of this approach for rational design of circular permutants.

### Study of the designed circular permutants in native conditions

**Fig. 3:**
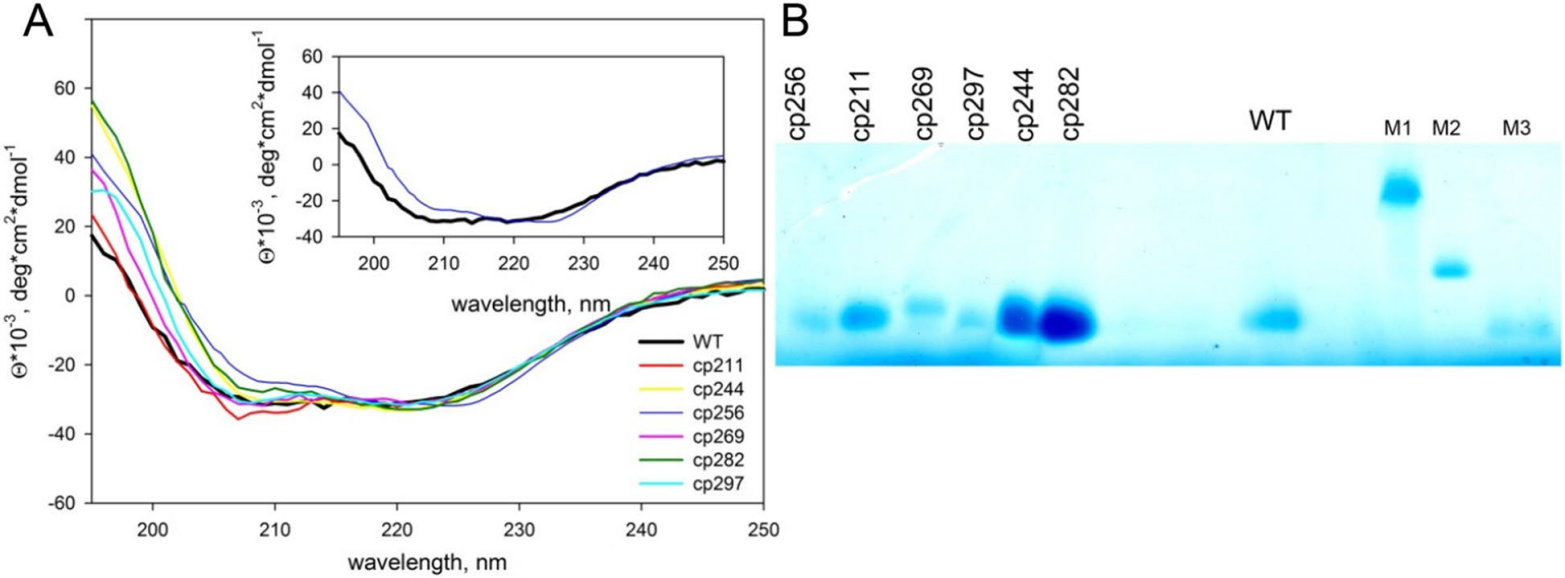
**A** Circular dichroism spectra of the GroEL apical domain (WT) and the designed circular permutants in the range of 195–250 nm. **B** Electrophoresis under native conditions for (from left to right) cp256, cp211, cp269, cp297, cp244, cp282, and WT. M1, M2, and M3 denote markers BSA, BCAB, and apomyoglobin, respectively

To experimentally investigate the influence of circular permutations on the secondary structure of AD-GroEL, we examined the far ultraviolet circular dichroism (CD) spectra of six designed circular permutants and the wild-type protein (Fig. 3). The spectra coincide in the 210–250 nm range, exhibiting two characteristic minima at 210 nm and 220 nm, typical of α-helical proteins. Based on the spectra, altering the positioning of the N- and C-termini had a minor impact on the protein's secondary structure under native conditions, except for the cp256 variant. The inset in Fig. 3A demonstrates that the minima at 210 nm and 220 nm are shifted for the cp256 variant compared to those in the wild-type protein spectrum, implying a different secondary structure for cp256. This alteration in secondary structure due to circular permutation appears to contribute to the observed reduced stability of the cp256 variant (see below).

Gel electrophoresis under native conditions (blue native electrophoresis; see Materials and Methods) reveals that all circular permutants exhibit comparable motility to the wild-type protein, with the exception of the cp269 variant, which displayed slightly reduced motility (Fig. 3B).

Consequently, based on the findings from experiments conducted under native conditions, we can infer that all designed circular permutants are appropriately folded, compact, and possess a secondary structure akin to that of the wild-type protein. Notably, the cp269 and cp256 variants exhibit minor deviations from the wild-type, showcasing differences in motility and secondary structure, respectively.

### Thermostability of the GroEL apical domain circular permutants

To experimentally assess the stabilities of the designed circular permutants, we investigated the protein structure's resilience to elevated temperatures using circular dichroism (CD) spectroscopy and differential scanning microcalorimetry (DSC).

**Fig. 4:**
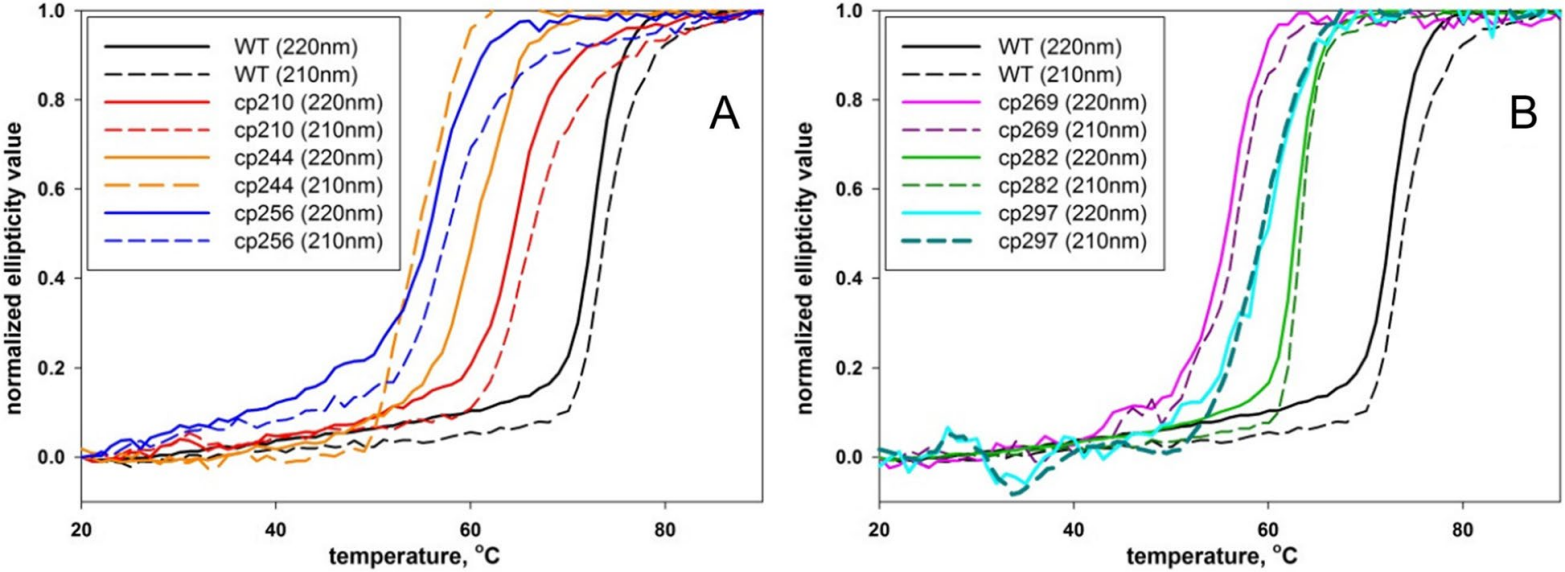
Dependence of ellipticity at 210 nm (dashed curves) and 220 nm (solid curves) on temperature **A** for GroEL apical domain WT, cp211, cp244, cp256 and **B** for GroEL apical domain WT, cp269, cp282, cp297. Ellipticity values are normalized for ease of comparison

Figure 4 illustrates the ellipticity changes at 210 nm and 220 nm in response to temperature variations. To facilitate comparisons across different proteins and wavelengths, the obtained curves were normalized within the 0 to 1 range. The wild-type AD-GroEL protein exhibited the highest level of thermal stability, with half-transition temperatures (t_1/2_) measured at 73.8 °C ± 0.3 °C and 72.4 °C ± 0.3 °C, as determined by CD measurements at 210 nm and 220 nm, respectively. These values slightly surpass the half-transition temperatures reported in previous studies for the GroEL apical domain, which were 57 °C [45, 46], 67 °C [35], and 70 °C [34].

**Fig. 5:**
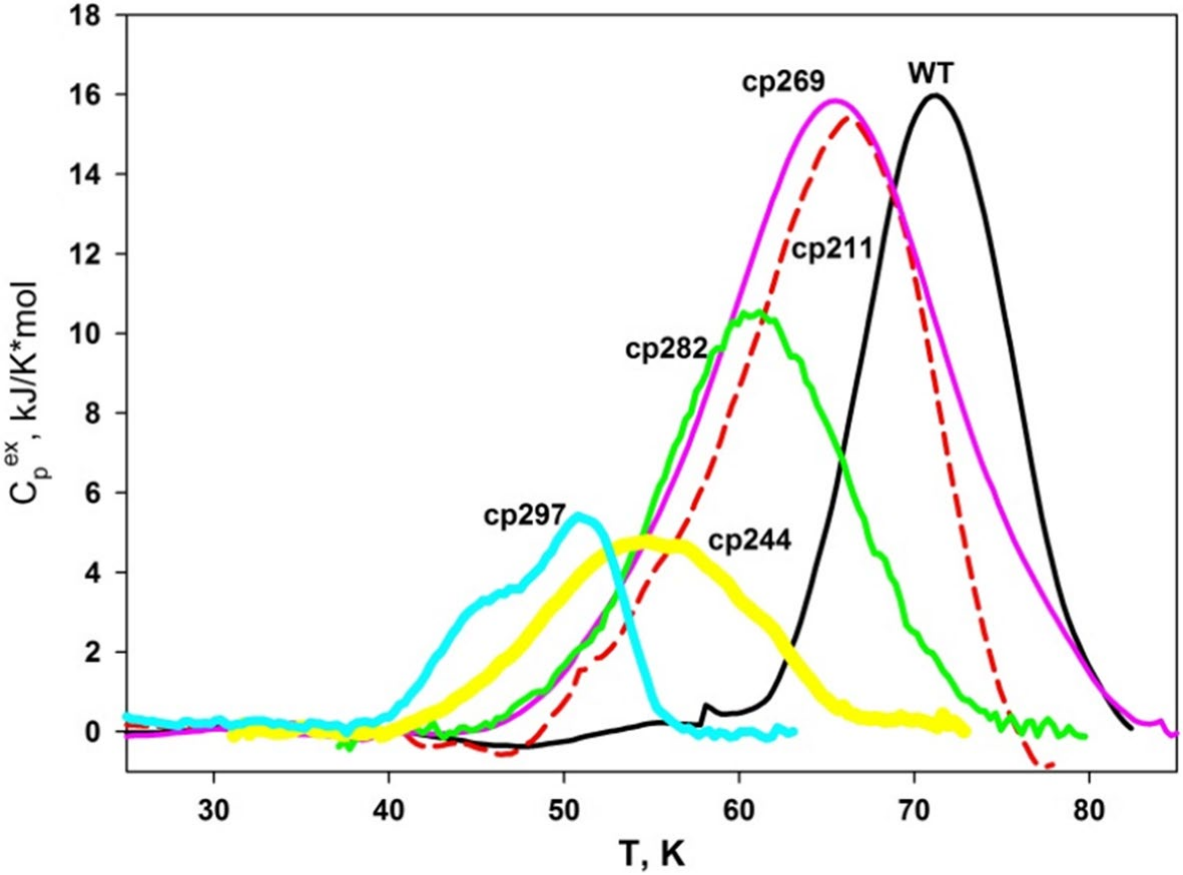
Temperature dependence of the excess heat capacity of the wild-type (WT) AD-GroEL protein and cp269, cp211, cp282, cp244, and cp297 circular permutants, respectively

Figure 5 depicts the temperature-dependent excess heat capacity of the circular permutants, as determined by DSC. Once more, the wild-type protein demonstrated the utmost stability, exhibiting the maximum temperature of the heat absorption peaks of proteins (t_m_) of 70.7 °C ± 0.1 °C, which notably concurs with the CD measurements.

However, the maximum temperature of the heat absorption peaks of proteins (t_m_) values obtained through the DSC method still exhibit disparities when compared to the half-transition temperatures (t_1/2_) acquired via the CD method (Table 1). Moreover, the CD measurements at both 210 nm and 220 nm display differences between each other as well (Fig. 4 and Table 1). These variations suggest the occurrence of intermediate states during the process of protein thermal denaturation.

**Table 1:** Heat denaturation parameters for the AD-GroEL protein and circular permutants obtained by different methods

	**PONDR**	**DSC t**_**m**_**, C (± 0.1)**	**CD Θ**_**210**_** t**_**1/2**_**,C (± 0.3)**	**CD Θ**_**220**_** t**_**1/2**_**,C (± 0.3)**
wt	0.03	71.1	73.8	72.4
cp211	0.31	66.2	66.4	64.3
cp244	0.40	54.9	54.5	60.4
cp256	0.66	—	57.6	55.6
cp269	0.23	65.5	56.6	55.5
cp282	0.38	61.1	63.4	62.7
cp297	0.69	45.1 and 51	59.2	59.8

PONDR—Intrinsic disorder score of specified amino acid residues predicted by PONDR® VLXT program

DSC t_m_—the maximum temperatures of the heat absorption peaks of protein obtained by the DSC method

CD Θ_210_ t_1/2_—the half-transition temperatures obtained by the CD ellipticity at 210 nm

CD Θ_220_ t_1/2_—the half-transition temperatures obtained by the CD ellipticity at 220 nm

Even for the wild-type protein, these discrepancies hold statistical significance. The emergence of intermediates in the case of the wild-type protein aligns with prior findings regarding the GroEL minichaperone (residues 191–376) [35]. For the designed circular permutants, the disparities are more conspicuous and indicate that they undergo transitional states during the heating process (as outlined in Table 1). Remarkably, for cp297, the temperature-dependent excess heat capacity curve exhibits two distinct peaks (see Fig. 5), providing additional evidence for the potential disruption of various protein domains or the formation of intermediate states during the protein's heat-induced denaturation [40].

In summary, our findings highlight the presence of intermediate states in the folding and unfolding processes, which complicates our study. Consequently, the verification of our hypothesis through multiple methods (CD at 210 nm, CD at 220 nm, and DSC) adds an extra layer of credibility to the results and demonstrates a good experimental practice. Given the intricacies observed in the (un)folding dynamics, it is interesting to extend our hypothesis testing to a two-state protein in future studies.

We should also note that our current work did not aim to study the features of thermal denaturation of each circular permutant in detail. Evidently, individual mutant proteins may manifest distinct intermediate states that could impact their aggregation characteristics or the formation of oligomers. Our primary aim was to establish a comparative analysis between disorder calculations and the outcomes of an empirical investigation into the thermal stability of the CPs. Consequently, we refrained from an exhaustive analysis of the folding and unfolding mechanisms underlying the designed circular permutants.

### Comparison of predicted parameters and experimentally measured thermal stability of circular permutants

**Fig. 6:**
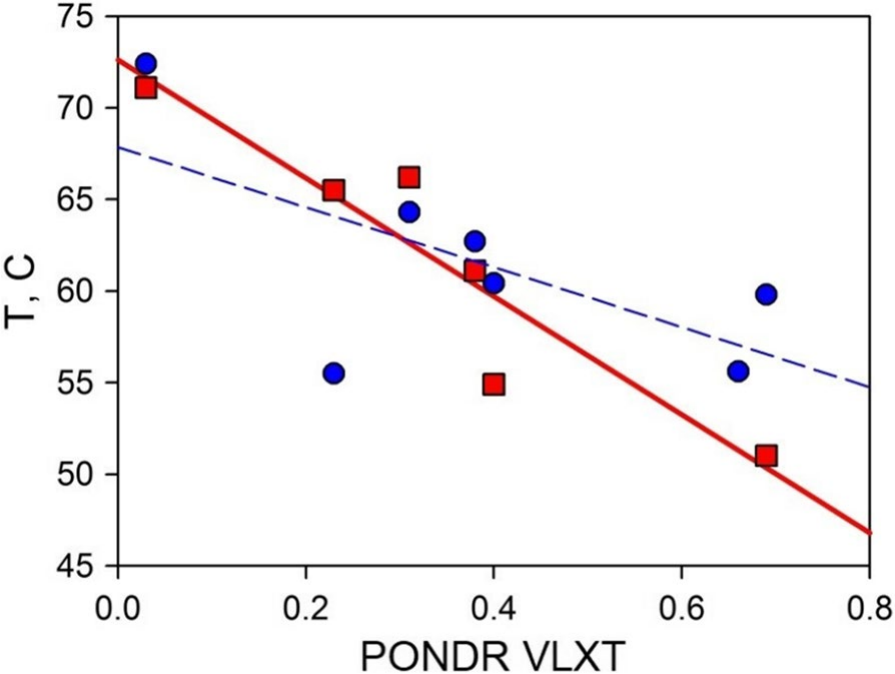
Dependence of the melting temperature of circular permutants, obtained by the CD at 220 nm (blue circles, Pearson correlation coefficient being -0.65) and by DSC for secondary peak of cp297 (red squares, Pearson correlation coefficient being -0.93), on the degree of disorder at the chain cleavage site

Figure 6 illustrates that the melting temperature for proteins, as determined by each of the methods, displays a reasonable inverse correlation with the PONDR® VLXT calculations (Table 1). The Pearson correlation coefficients stand at -0.62 for CD at 210 nm, -0.65 for CD at 220 nm, -0.93 for DSC, when we consider the main peak of cp297 with T_m_ = 51.0°C, and -0.95 for DSC, when we consider the second peak of cp297 with T_m_ = 45.1°C. However, a distinct variation in slope across the data obtained through different methods is observed. This disparity is to be anticipated for proteins that undergo multi-state transitions involving intermediate states during the unfolding process. Nevertheless, Fig. 6 demonstrates that, for our purposes, both dependencies yield satisfactory results. In fact, to identify the most stable or most destabilized circular permutant variant, we can effectively accomplish this computationally by relying on PONDR® VLXT's disorder predictions. Consequently, the evaluation of local instability, which exhibits a strong inverse correlation with protein disorder predictor calculations, offers a viable approach for the design of circular permutants in globular proteins.

One should keep in mind though that making correlations based just on 6 data points does not provide any strong indication towards any conclusion. In fact, to have a possibility to reliably discuss statistically significant correlations, one should study very large number of mutant proteins. However, even if 100 proteins are analyzed, it would not be possible to establish a strong correlation if the proteins are characterized by different structures. This is because, it can always be said that the replacement of amino acid residues in the α-helix is not the same as amino acid substitution in the loop or in β-structure. Therefore, the question of how to increase the number of experimental points in Fig. 6 is not a simple task. We tried to solve this problem by studying proteins with different amino acid sequences but identical structures, ribosomal proteins L1 from the halophilic archaeon *Haloarcula marismortui* (HmaL1) and extremophilic bacterium *Aquifex aeolicus* (AaeL1) [23]. This analysis revealed that the protein melting temperature was not changed when a disulfide bond was introduced into the predicted structured region in AaeL1, whereas introduction of a disulfide bond introduced into the same region that was predicted as a weakened in HmaL1, resulted in the noticeable increase in the protein thermal stability [23]. The positive results of that study indicated that, in principle, it is possible to use PONDR® VLXT outputs for designing mutant proteins with increased stability. Hence, in the present study we do not answer the question whether there is a correlation between the melting temperatures for the proteins and the degree of disorder at the chain cleavage site. Instead, we show that our approach works not only when introducing disulfide bridges [21–24] but also when designing circular permutants.

### Using AlphaFold to analyze the structure and stability of circular permutants

AlphaFold has gained widespread popularity for its remarkable ability to accurately predict the native 3D structures of proteins [47]. Motivated by this achievement, we opted to harness its capabilities to dissect disparities between the designed AD-GroEL circular permutants and the wild-type protein. We hoped to gain valuable structural insights into our findings by predicting alterations in the CPs' structures. Given the consistent reduction in T_m_ values across all CPs (Table 1), we anticipated a logical relationship where the greater destabilization corresponds to more pronounced deviations from the wild-type protein structure.

**Fig. 7:**
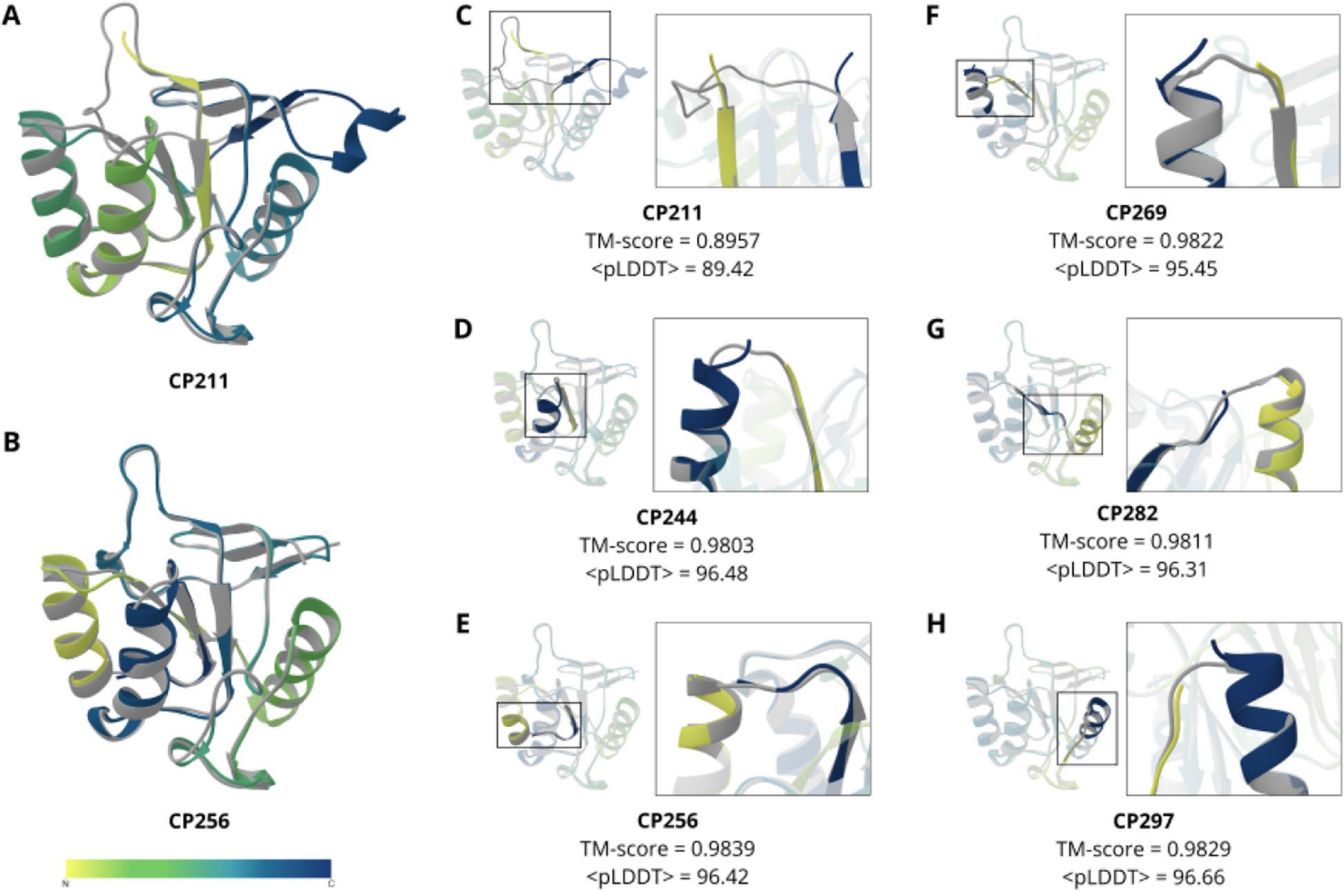
3D structure of AD-GroEL (PDB: 1DER, shown in grey) aligned with the structures of the circular permutants predicted by the AlphaFold program (colored with a color gradient located in the bottom of Figure). The wild-type protein structure is shown in grey. The orientation of the structures is the same in all subfigures. **A** Structure of AD-GroEL and cp211 circular permutant (least differing structures). **B** Structure of AD-GroEL and circular permutant cp256 (most differing structure). **C**-**G** – structural alignment of the six circular permutants with wild-type protein structure. The insets depict new N- (blue) and C- (green) terminal regions for each circular permutant. The orientation in the insets is optimized for a better view

However, we observed a somewhat contradictory scenario. Structural alignments revealed the greatest divergence in structure within the most stable cp211 variant (Fig. 7B), while the least notable deviation was detected in the highly destabilized cp256 protein (Fig. 7A). Notably, we performed structural alignment using the sequence order-unaware mode of the TM-align program [48], ensuring that structural differences are not attributable to differing arrangements of structural fragments. For cp211, it is plausible to consider that its altered structure might be somehow less destabilized, although this remains improbable but conceivable. In contrast, the nearly identical structure of cp256 predicted by AlphaFold offers no clear explanation for the substantial ~ 16°C reduction in melting temperature (Table 1). Furthermore, skepticism toward the AlphaFold-predicted structure of cp256 is further compounded by its secondary structure, which evidently deviates experimentally from that of the wild-type and other CPs (Fig. 3A), while inexplicably coinciding with the AlphaFold prediction.

We also lack explanations for the diminished mobility of cp269 as compared to the AlphaFold-predicted structure (Fig. 7F). The disparities from the wild-type protein structure are exceedingly minute, conflicting with the reduced compactness identified in the experimental data (Fig. 3B). Similarly, alterations in the AlphaFold confidence scores fail to account for the varying stability among circular permutants (Table 1), aligning with prior observations [49].

As depicted in Fig. 7, it appears that AlphaFold does not discern between circular permutants, despite experimental evidence highlighting distinctions among the proteins (Figs. 3, 4, and 5). This implies that AlphaFold recognizes the shared spatial configuration of amino acids as presented in PDB structures, rather than individually predicting the 3D structure for each mutant. Consequently, AlphaFold adeptly predicts the overarching attributes of protein structure with the requisite accuracy crucial for diverse structural bioinformatics tasks [50, 51], yet falls short in capturing local alterations within these structures. This observation harmonizes with previous findings concerning single-point mutations [49, 52]. This perspective also signifies that AlphaFold draws on the statistics derived from PDB and multiple sequence alignments, without assimilating the physics underlying the protein folding [53, 54].

Overall, circular permutations present a suitable test case for evaluating AlphaFold. Given that circular permutation of substantial segments of the amino acid sequence preserves the relative arrangement of residues in three-dimensional space, AlphaFold retains the ability to discern the 3D structure of the wild-type protein, seemingly using PDB data. Nonetheless, AlphaFold's predictions appear to adhere too closely to the wild-type protein's structure, leading to an underestimation of the alterations resulting from mutations. This agrees with findings from a prior investigation [52].

### PONDR® VLXT vs. PONDR-FIT

The intrinsic disorder score calculations shown in Figs. 1 and 2 were conducted using the PONDR® VLXT algorithm (http://www.pondr.com) [41], and the AD-GroEL circular permutants were designed based on these calculations. In our previous studies, we hypothesized that if a region of a globular protein is predicted by a program as intrinsically disordered but the structure of this region is known, this may serve as an indication of the structural “weakness” of this region. Therefore, the results of the per-residue evaluation of intrinsic disorder predisposition allows the detection of “weak” and “stabilized” parts of a protein, despite the fact that they are all structured. We tested this hypothesis on several proteins. For example, using this principle, the stabilizing cysteine bridges were designed in the GαO and ribosomal protein L1 [21, 23].

**Fig. 8:**
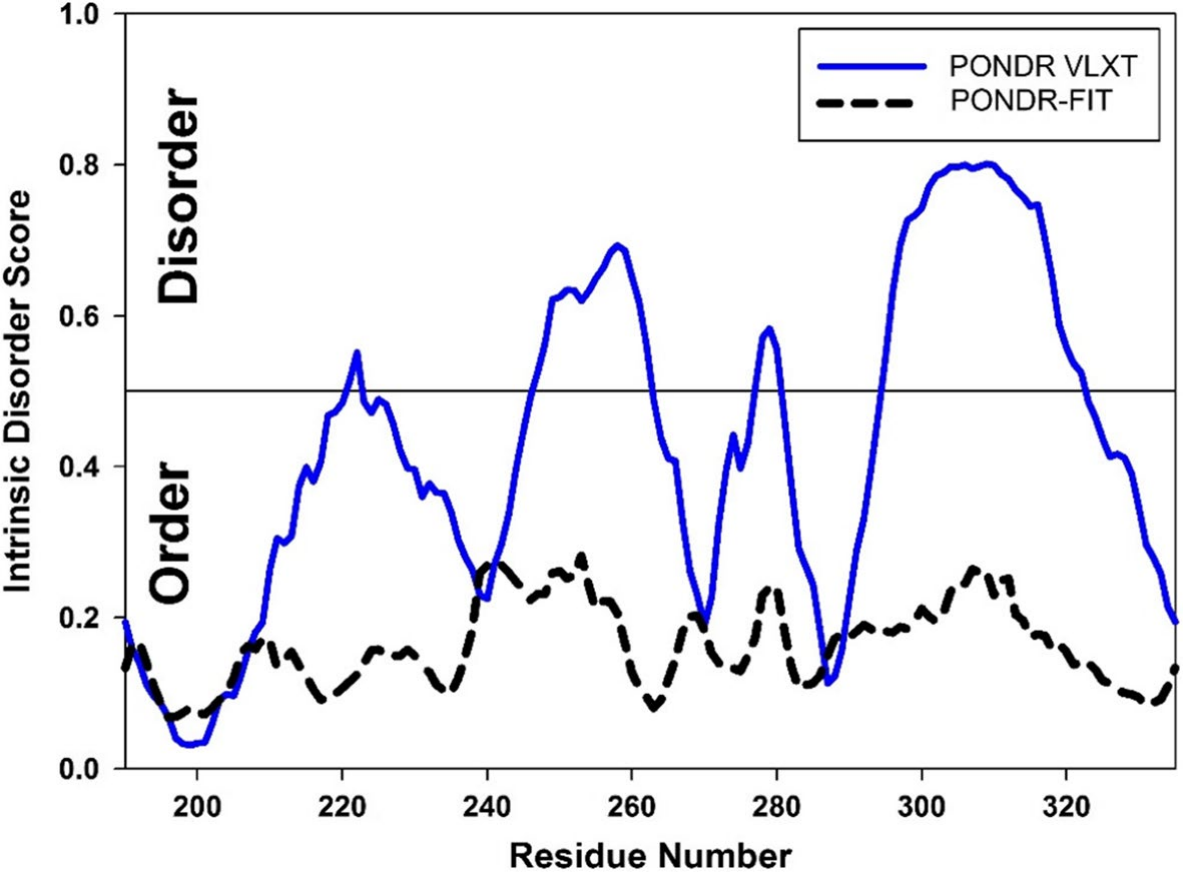
Intrinsic disorder profiles of AD-GroEL generated by the PONDR® VLXT and PONDR-FIT algorithms

In our studies, the intrinsic disorder predictor PONDR® VLXT is typically used, as this algorithm is very sensitive to the local sequence features and peculiarities [41, 55]. This tool was one of the first (if not the first) per-residue disorder predictors and serves as the originator of the family of PONDR predictors, each developed for some specific purposes, with the most recent being a metapredictor PONDR-FIT, which is a consensus artificial neural network prediction method developed by combining the outputs of several individual disorder predictors (including PONDR® VLXT) [18]. Not surprisingly, a researcher looking for the PONDR program will find this PONDR-FIT algorithm that is freely available at (http://original.disprot.org/pondr-fit.php). Although to an inexperienced user, it may seem that the PONDR® VLXT and PONDR-FIT are the same program, in reality, these tools give completely different results. This is illustrated by Fig. 8 that shows a comparison of the calculations performed for the apical domain of GroEL (AD-GroEL) by these two programs. It can be seen that the PONDR-FIT program indicates the absence of weakened regions in the amino acid sequence of AD-GroEL. If we had used PONDR-FIT calculations when the current project was started, we would not have planned to work with the apical domain of GroEL, since, according to the disorder profile shown in Fig. 8, mutations in different regions of the amino acid sequence will affect the protein in the same way.

A completely different assumptions can be made based on the outputs of the PONDR® VLXT algorithm. In fact, the curve generated by the PONDR® VLXT program (Fig. 8) has several characteristic minima and maxima. According to this profile, mutations in different regions of the protein are expected to have different effects on the AD-GroEL stability. Therefore, this assumption was used to plan our study, the final results of which are illustrated by the correlation shown in Fig. 6. Of course, the generality of this hypothesis can be questioned, since the peculiarities of this correlation will depend on the structure of the protein under study and on the complexity of the mutations planned. In difficult cases, instead of looking for exact correlations, one can simply use the division of proteins into stable and unstable regions. For example, based on Fig. 2, it can be assumed that the cp211 and cp269 mutants will be more stable than cp256 and cp297. And this assumption turned out to be correct (Fig. 5, Table 1).

Unfortunately, neither correlation nor even division into stable and unstable regions is possible if one would use the outputs of the PONDR-FIT for the analysis. This is an interesting observation, as, overall, PONDR-FIT was found to improve the prediction accuracy compared to the single predictors [18]. However, the detailed comparison of the performance of the PONDR® VLXT and PONDR-FIT algorithms is beyond the scope of this study. Especially because we used disorder predictors for the non-standard purpose of searching for weakened and stabilized regions of a fully structured protein. The main purpose of this work is to help experimentalists by proposing some useful ideas, such as using the PONDR® VLXT algorithm in the design of stable proteins [22].

### Published studies on S6 permutants support the applicability of the disorder-based design of permutations

As it was already pointed out, numerous studies have concentrated on the design of linkers for circularly permuted proteins [14–16], leading to a very important conclusion that the specific features of a new linker region might have very significant effects on the permuted protein stability. In fact, it was pointed out that the circular permutation involves two partly compensating energetic components, an energetic penalty from cutting up the polypeptide chain in a new position and a structural stabilization due to the linkage of the N- and C- termini [56]. Therefore, it might be expected that the peculiarities of the linker region design might affect the protein stability. The validity of this statement can be checked by the analysis of the published experimental data on the conformational stabilities of the small ribosomal subunit protein S6 permuteins with the same cleavage sites but different linkers [56–58]. Hadlug et al. (2008) generated permuteins by the P^13−14^, P^33−34^, P^54−55^, P^68−69^, and P^81−82^ incisions within the loops linking the secondary structure elements of S6, whereas the linkage of the wild-type ends was achieved via the removal of ten residues at C-terminal end (KSQEPFLANA) and two residues at the N-terminal end (MR) of S6 sequence and connection of the modified using a loop consisting of the amino acid residues ASTTPG [56]. Note that the S6 permuteins with the P^13−14^ and P^68−69^ incisions and the same design of a linker region were analyzed in earlier study of this group [58]. In the Miller et al. (2002) study, residues 14, 36, and 55, which are located within the loop regions (i.e., P^13−14^, P^35−36^, and P^54−55^ incisions), were selected as new N termini, whereas old N- and C-termini were connected by a simple AGA linker [57]. Both groups analyzed peculiarities of the guanidinium chloride (GdmCl)-induced unfolding-refolding processes of the resulting S6 permuteins in comparison with those of the wild type of this protein. Therefore, since these two teams used different linkages, and since three of the studied S6 permutants had same or similar cleavage sites, the corresponding S6 permuteins represent an important system that can provide an answer on the actual effects of the linkages on the permutant stability.

According to Miller et al. (2002), based on their ΔG_u_, analyzed wild type (WT) and permuted proteins can be arranged in the following order [57]:

$$\mathrm{P}^{13-14}\left(4.1\pm0.1\,\text{kcal mol}^{-1}\right)<\mathrm{P}^{35-36}\left(5.4\pm0.3\,\text{kcal mol}^{-1}\right)<\text{WT}\left(8.5\pm0.5\,\text{kcal mol}^{-1}\right)<\mathrm{P}^{54-55}\left(9.1\pm0.5\,\text{kcal mol}^{-1}\right)$$

Based on the results reported by the Oliveberg’s group, the WT S6 and its permutants can be arranged based their ΔG_D−N_ values as follows [56, 58]:

$$\mathrm{P}^{13-14}\left(3.89/4.29\text{kcal mol}^{-1}\right)<\mathrm{P}^{81-82}\left(5.15\,\text{kcal mol}^{-1}\right)<\mathrm{P}^{33-34}\left(5.76\,\text{kcal mol}^{-1}\right)<\mathrm{68-69}\left(6.82/6.91\text{kcal mol}^{-1}\right)<\text{WT}\left(8.22/8.97\,\text{kcal mol}^{-1}\right)<\mathrm{P}^{54-55}\left(9.36\,\text{kcal mol}^{-1}\right)$$

Therefore, since no major differences were detected for the permutants with the similar cleavage sites (P^13−14^, P^35−36^/ P^33−34^, and P^54−55^ incisions) but different linkers, the results of these studies indicated that the linker did not have a major role in the permutant stability (at least in the context of the S6 system).

**Fig. 9:**
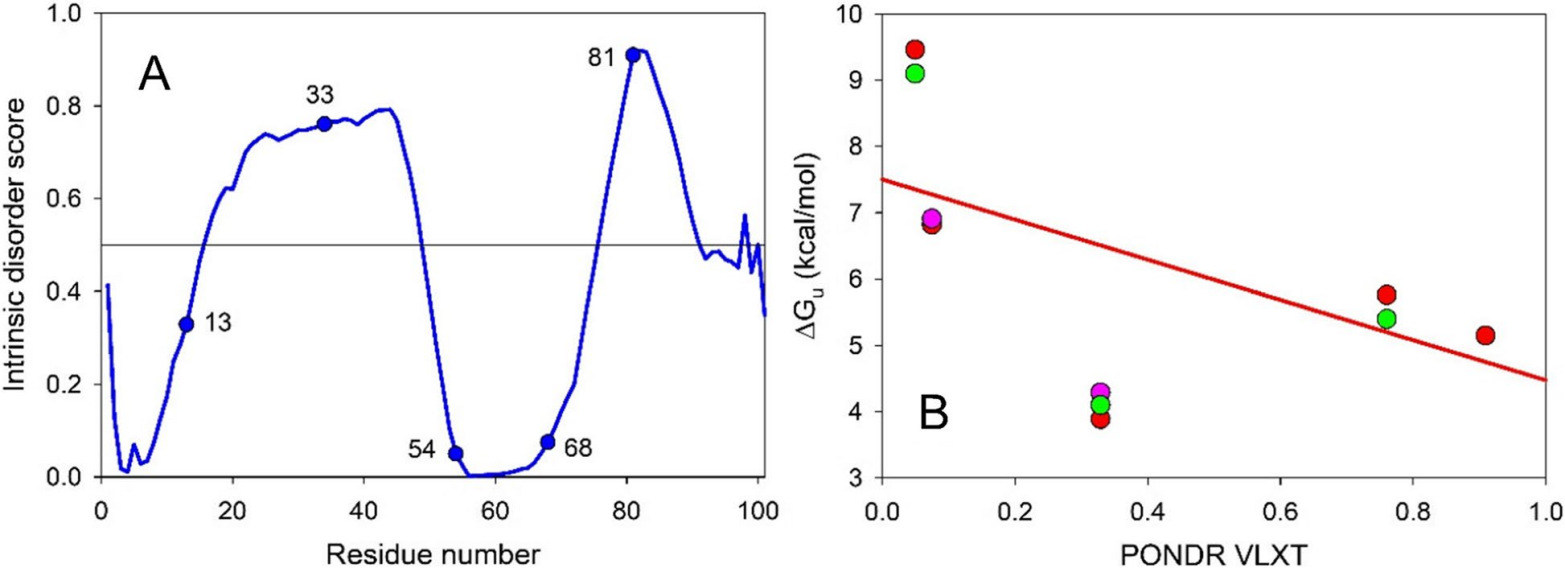
**A** The PONDR® VLXT-based evaluation of the per-residue intrinsic disorder propensity of the ribosomal protein S6 from *Thermus thermophiles* (UniPrit ID: P23370). The circles denote the position of the sites of the designed breaks in the amino acid sequence. **B** Dependence of the changes in the global stability (ΔG_u_) of S6 circular permutants, determined from the analysis of the guanidinium chloride-induced unfolding of the analyzed proteins on the local intrinsic disorder propensity of the designed cleavage cites in the wild type protein. Data were retrieved from Haglund et al. [56] (red circles), Lindberg et al. [58] (pink circles), and Miller et al. [57] (green circles). Pearson correlation coefficient is -0.57, reflecting a moderate negative correlation, which means there is a tendency for high PONDR® VLXT scores to go with low ΔG_u_ values

On the other hand, data reported by these groups provided an important support to our concept that the peculiarities of the PONDR® VLXT-generated intrinsic disorder profile can be used in the rational design of the cleavage sites in permutants. This conclusion is illustrated by Fig. 9A showing the localization of the permutation sites within the disorder profile of the S6 protein from *Thermus thermophiles*. It is clearly seeing that the locations of the P^13−14^, P^33−34^, P^54−55^, P^68−69^, and P^81−82^ incisions are characterized by a very noticeable variability of the local intrinsic disorder predisposition. Furthermore, Fig. 9B shows that in line with our data for the AD-GroEL permutants, stability of the S6 permuteins is inversely correlated with the local disorder propensity of cleavage site. Therefore, these data provide further evidence that, in principle, it is possible to use PONDR® VLXT outputs for designing permutants with the increased stability. Importance of this conclusion is further stringent by the fact that it is based on the analysis of the conformational stability of permutants of two different proteins against temperature-induced denaturation (AD-GroEL) or GdmCl-unfolding (S6).

## Conclusions

Selecting the optimal points for new N- and C-termini stands as a key factor in the protein redesign process through circular permutations. Building upon our previous achievements in applying disorder predictions to engineer stabilizing disulfide bonds in GFP and GαO proteins [20, 21] we harness disorder-related insights in this investigation to guide the selection of new N- and C-termini locations for circular permutations within the GroEL apical domain. Utilizing the set of glycines positioned within loops as potential cleavage sites, we substantiate our hypothesis that higher disorder values correspond to decreased stability in circular permutants. Experimental assessments of melting temperatures through circular dichroism and differential scanning microcalorimetry align remarkably well with disorder predictions made by the PONDR® VLXT algorithm [18, 41].

Obviously, linkers are very important when designing stable circular permutants. Many articles are devoted to the design of universal linkers [59, 60], and to design linkers in a particular protein, different methods can be used ranging from a simple enumeration of the linker length and its amino acid composition to conducting molecular dynamics simulation and using the machine learning [61–63]. However, in this article, we did not consider the question of how the linker might affect the designed circular permutants. Instead, we focused on elaborating means for finding the suitable cleavage sites for generating new N- and C-termini. As a result, we have chosen the simplest linker, realizing in advance that it might not be the most effective. We believe that finding the "right" linker, which will further improve the stability of the protein, represents the next task after finding the “right” cleavage points.

Concluding, our study introduces the first systematic computational approach to rationally choose cleavage points in the designing circular permutants with targeted stability. We hope that this work will streamline and enhance the efficiency of protein design via circular permutations.
